# Risk factors and an early predictive model for Kawasaki disease shock syndrome in Chinese children

**DOI:** 10.1186/s13052-024-01597-x

**Published:** 2024-02-03

**Authors:** Mingming Zhang, Congying Wang, Qirui Li, Hongmao Wang, Xiaohui Li

**Affiliations:** 1 Department of Cardiology, Children’s Hospital Capital Institute of Pediatrics, Beijing, 10020 China; 2 Department of Cardiology, Capital Institute of Pediatrics-Peking University Teaching Hospital, Beijing, China; 3 Department of Cardiology, Beijing Children’s Hospital, Capital Medical University, National Centre for Children’s Health, Beijing, China

**Keywords:** Mucocutaneous Lymph Node Syndrome, Shock, Complications, Risk factors, Nomograms

## Abstract

**Background:**

Kawasaki disease shock syndrome (KDSS), though rare, has increased risk for cardiovascular complications. Early diagnosis is crucial to improve the prognosis of KDSS patients. Our study aimed to identify risk factors and construct a predictive model for KDSS.

**Methods:**

This case-control study was conducted from June, 2015 to July, 2023 in two children’s hospitals in China. Children initially diagnosed with KDSS and children with Kawasaki disease (KD) without shock were matched at a ratio of 1:4 by using the propensity score method. Laboratory results obtained prior to shock syndrome and treatment with intravenous immunoglobulin were recorded to predict the onset of KDSS. Univariable logistic regression and forward stepwise logistic regression were used to select significant and independent risk factors associated with KDSS.

**Results:**

After matching by age and gender, 73 KDSS and 292 KD patients without shock formed the development dataset; 40 KDSS and 160 KD patients without shock formed the validation dataset. Interleukin-10 (IL-10) > reference value, platelet counts (PLT) < 260 × 10^9^/L, C-reactive protein (CRP) > 80 mg/ml, procalcitonin (PCT) > 1ng/ml, and albumin (Alb) < 35 g/L were independent risk factors for KDSS. The nomogram model including the above five indicators had area under the curves (AUCs) of 0.91(95% CI: 0.87–0.94) and 0.90 (95% CI: 0.71–0.86) in the development and validation datasets, with a specificity and sensitivity of 80% and 86%, 66% and 77%, respectively. Calibration curves showed good predictive accuracy of the nomogram. Decision curve analyses revealed the predictive model has application value.

**Conclusions:**

This study identified IL-10, PLT, CRP, PCT and Alb as risk factors for KDSS. The nomogram model can effectively predict the occurrence of KDSS in Chinese children. It will facilitate pediatricians in early diagnosis, which is essential to the prevention of cardiovascular complications.

**Supplementary Information:**

The online version contains supplementary material available at 10.1186/s13052-024-01597-x.

## Background

Kawasaki disease shock syndrome (KDSS) is a severe manifestation of Kawasaki disease (KD) characterized by hemodynamic instability before the infusion of intravenous immunoglobulin (IVIG), with a complex etiology [1]. The incidence of KDSS ranges from 0.9% to 5.3% of KD cases and has been increasing year by year using the national or regional databases [2, 3].

Multiple studies have revealed, compared with KD patients with hemodynamically normal status, patients with KDSS have increased risk for cardiovascular complications [4–6]. Gámez-González et al. reported that 20% (3/11) of patients with KDSS developed giant coronary aneurysms, and 27% (2/11) presented myocardial infarction [7]. It is known to all that early diagnosis and timely administration of IVIG are crucial to improve the prognosis of KD patients [8]. However, since the symptoms of KDSS patients are not typical and often involve multiple organs and systems, the diagnosis relies on rich clinical experience and careful observation of clinical signs and symptoms [9–11]. It would be of great significance if objective laboratory indicators involved in the KDSS could be early identified.

Park et al. found that patients with KDSS had a significantly older age, lower hemoglobin (Hgb), lower platelet (PLT), higher C-reactive protein (CRP), higher serum creatinine level (Scr) and lower albumin (Alb) than patients with KD [12]. Qiu et al. demonstrated that the absolute neutrophil count (ANC), alanine aminotransferase (ALT), aspartate aminotransferase (AST), and CRP were higher in KDSS patients than in KD patients [13]. However, Chen et al. revealed that there were no significant differences in ALT and AST between KDSS patients and KD patients [14]. Although the conclusions of the prior studies were not the same, they have provided clues to the risk profiles of KDSS. Considering the pathogenesis of KDSS may involve a decrease in peripheral vascular resistance, myocarditis and capillary leakage [1, 15–17], it remains challenging to use a single indicator to predict the occurrence of KDSS in clinical practice.

In this study, we summarized the laboratory results of KDSS patients in two children's hospitals in Beijing to identify risk factors associated to KDSS. For better clinical application, we constructed a comprehensive nomogram model for KDSS among Chinese children.

## Methods

### Study patients

Children who were initially diagnosed with KD in the Children’s Hospital Capital Institute of Pediatrics and Beijing Children’s Hospital from June, 2015 to July, 2023 were enrolled. We used the propensity score matching by age and gender to pair KDSS patients and KD patients without shock syndrome in a ratio of 1:4.

The eligible criteria were based on the American Heart Association Guidelines and Kanegaye criteria [1, 18]. The exclusion criteria were as follows: 1) Combined with sepsis shock or other cardiovascular, hypotension diseases; 2) Incomplete clinical records.

The conduct of this study was approved by the Ethics Committee of the Capital Institute of Pediatrics (Approval Number SHERLL2023007).

### Data collection

We collected age, gender and laboratory results which were conducted prior to the onset of KDSS and treatment with IVIG. Laboratory results including white blood cell (WBC), ANC, PLT, Hgb, CRP, procalcitonin (PCT), erythrocyte sedimentation rate (ESR), ALT, AST, Scr, Alb, interleukin-6 (IL-6), interleukin-8 (IL-8) and interleukin-10 (IL-10) were shared in both hospitals. If the laboratory measurements were repeated, we recorded the earliest indexes for further analyses.

### Serum cytokines detection

Patients were tested for IL-6, IL-8 and IL-10 at admission before the onset of KDSS and treatment with IVIG. Serum cytokine concentrations were measured by chemiluminescence (Siemens Immulite 1000 chemiluminescence analyzer) in the Children’s Hospital Capital Institute of Pediatrics, where the reference ranges of IL-6, IL-8 and IL-10 were 0–3.4 ng/L, 0–62 ng/L, 0–9.1 ng/L, respectively. Concentrations of the three cytokines were measured by using the fluorescence-activated cell sorting (BD FACSCalibur™ Flow Cytometry instruments) in the Beijing Children’s Hospital, where the reference ranges of IL-6, IL-8, IL-10 were ≤ 5.4 ng/L, ≤ 20.6 ng/L, ≤ 12.9 ng/L, respectively.

### Statistical analysis

Kolmogorov–Smirnov test was used to test the normality of continuous variables. Depending on whether the parameters were in Gaussian distributions, continuous variables are reported as median (interquartile ranges) or mean ± standard deviation, and between-group differences were compared using Two-sample Student’s t-test or Mann–Whitney test. Categorical variables are expressed as numbers (percentage) and compared using χ^2^ test. A two-sided *P* value < 0.05 was considered statistically significant.

Spearman correlation coefficients were used to quantify relationship between the laboratory results. Univariable logistic regression and forward stepwise logistic regression were used to select significant and independent risk factors for KDSS. To facilitate clinical application, continuous variables were transformed into dichotomous variables. The value of IL-10 was normalized by using the clinical reference values. The optimal cut-off values of PLT, CRP, PCT and Alb were chosen according to the maximum Youden’s index. Finally, we used multivariate logistic regression to construct the predictive model. A nomogram plot was used to visually illustrate this model.

Internal validation was performed by using the bootstrapping method with 1000 repetitions. The receiver operator characteristic curve (ROC) and the calibration curve analysis were used to evaluate the discrimination and calibration of the predictive model in both development and case-independent validation datasets. Sensitivity, specificity, and the area under the receiver operating characteristic (AUC) were calculated. Decision curve analysis (DCA) was used to evaluate the net benefits of the predictive model. All statistical analyses were performed using the R software version 4.2.3 and IBM SPSS Statistics 27.0.1 software.

## Results

### Demographic information and laboratory data of patients

During the study period, a total of 3086 patients who were initially diagnosed with KD were included; 459 were excluded for missing clinical data; leaving 2627 in the final analysis. There were 2587 KD patients from the Children’s Hospital Capital Institute of Pediatrics, of whom 73 (2.8%) were KDSS. KDSS patients were older than KD patients without shock [3.57 ± 2.16 vs. 2.34 ± 1.59, *P* < 0.001], while no statistically significant difference in sex distribution was observed between the two groups [boys: 37 (50.7%) vs. 1539 (61.2%), *P* = 0.09]. There were 40 KD patients from the Beijing Children’s Hospital, and they met our inclusion and exclusion criteria for the KDSS. The average age was 3.59 (interquartile range: 2.42, 5.73) years and 26 (65%) were boys.

After propensity scores matching by age and gender, a dataset comprising 73 KDSS patients and 292 KD patients without shock syndrome from the Children’s Hospital Capital Institute of Pediatrics was formed for analyzing the risk factors and constructing a predictive model. Additionally, another dataset consisting of 40 KDSS patients from the Beijing Children’s Hospital and 160 KD patients without shock from the Children’s Hospital Capital Institute of Pediatrics was formed to validate this model. Demographic information and laboratory results between the two groups in development and case-independent validation datasets are presented in Table 1 and Supplemental Table 1, respectively.

**Table 1:** Comparison with demographic information and laboratory data between the two groups after propensity score matching in the development dataset

Variables	KDSS group (*n* = 73)	KD group (*n* = 292)	*P* values
Age, years	3.42 (1.58, 4.58)	3.17 (1.58, 4.42)	0.59
Gender (male)	37 (51)	138 (47)	0.69
WBC, × 10^9^/L	15.04 (11.48, 20.77)	13.11 (10.48, 16.34)	< 0.01
ANC, × 10^9^/L	12.5 (7.97, 17.55)	8.36 (6.21, 11.98)	< 0.01
PLT, × 10^9^/L	259 (169, 362)	362.5 (296, 435.25)	< 0.01
Hgb, g/L	108 (99, 117)	111 (105, 118)	0.17
CRP, mg/dl	115 (81.39, 164)	55.7 (25, 87)	< 0.01
PCT, ng/mL	4.62 (2.27, 10.93)	0.5 (0.21, 0.92)	< 0.01
ESR, mm/60 min	61 (40, 83)	68.5 (49, 85)	0.03
ALT, U/L	38.1 (18, 68.6)	20.35 (13.55, 57.92)	< 0.01
AST, U/L	28.4 (20, 45.7)	26 (21.58, 34.42)	0.36
Scr, μmol/L	31.9 (23.5, 43.4)	26.2 (22.78, 31.7)	< 0.01
Alb, g/L	31.4 (27.3, 33.4)	35.45 (32.6, 38.12)	< 0.01
IL-6, pg/ml	83.4 (26.90 248)	22 (7.98, 54.32)	< 0.01
IL-8, pg/ml	23.8 (14, 44)	19.05 (10.8, 47.25)	0.65
IL-10, pg/ml	51.5 (11.8, 115)	6.61 (5, 14.7)	< 0.01

*KDSS* Kawasaki disease shock syndrome, *KD* Kawasaki disease, *WBC* White blood cell, *ANC* Absolute neutrophil count, *PLT* Platelet, *Hgb* Hemoglobin, *CRP* C-reactive protein, *PCT* Procalcitonin, *ESR* Erythrocyte sedimentation rate, *ALT* Alanine aminotransferase, *AST* Aspartate aminotransferase, *Scr* Serum creatinine, *Alb* Albumin, *IL-6* Interleukin-6, *IL-8* Interleukin-8, *IL-10* Interleukin-10. Gender is expressed as number (percentage), while the other variables are expressed as median with quartile ranges

### Selection of significant factors

As shown in Table 2, 9 candidate variables: WBC, ANC, PLT, CRP, PCT, ESR, Scr, Alb, IL-10 were associated with the risk of KDSS. We removed ANC from the further analysis because high correlation was found between WBC and ANC (Spearman’s r = 0.9; *P* < 0.001). By using the forward stepwise logistics regression, PLT, CRP, PCT, Alb and IL-10 were teased out as significant and independent risk factors of KDSS (Table 2).

**Table 2:** Identification of significant risk factors associated to KDSS

Variables	Univariable logistic regression		Multivariable logistic regression*	
	OR (95% CI)	*P* values	OR (95% CI)	*P* values
WBC, × 10^9^/L	1.076 (1.035–1.118)	< 0.001		
ANC, × 10^9^/L	1.123 (1.077–1.172)	< 0.001		
PLT, × 10^9^/L	0.992 (0.990–0.995)	< 0.001	0.995 (0.992–0.998)	0.002
Hgb, g/L	1.003 (0.990–1.016)	0.667		
CRP, mg/dl	1.020 (1.015–1.026)	< 0.001	1.012 (1.004–1.019)	0.002
PCT, ng/mL	1.373 (1.255–1.501)	< 0.001	1.180 (1.083–1.285)	< 0.001
ESR, mm/60 min	0.988 (0.977–0.998)	0.016		
ALT, U/L	1.002 (0.999–1.005)	0.256		
AST, U/L	1.002 (0.999–1.006)	0.176		
Scr, μmol/L	1.068 (1.040–1.096)	< 0.001		
Alb, g/L	0.816 (0.767–0.869)	< 0.001	0.888 (0.817–0.965)	0.005
IL-6, ng/L	1.003 (1.002–1.004)	< 0.001		
IL-8, ng/L	0.997 (0.993–1.000)	0.073		
IL-10, ng/L	1.020 (1.013–1.027)	< 0.001	1.011 (1.004–1.018)	0.001

*OR* Odds ratio, *95%CI* 95% confidence interval, *KDSS* Kawasaki disease shock syndrome, *KD* Kawasaki disease, *WBC* White blood cell, *ANC* Absolute neutrophil count, *PLT* Platelet, *Hgb* Hemoglobin, *CRP* C-reactive protein, *PCT* Procalcitonin, *ESR* Erythrocyte sedimentation rate, *ALT* Alanine aminotransferase, *AST* Aspartate aminotransferase, *Scr* Serum creatinine, *Alb* Albumin, *IL-6* Interleukin-6, *IL-8* Interleukin-8, *IL-10* Interleukin-10. ※ Variables are selected by using univariate logistic regression and forward stepwise regression analyses

### Development of the predictive nomogram

**Fig. 1:**
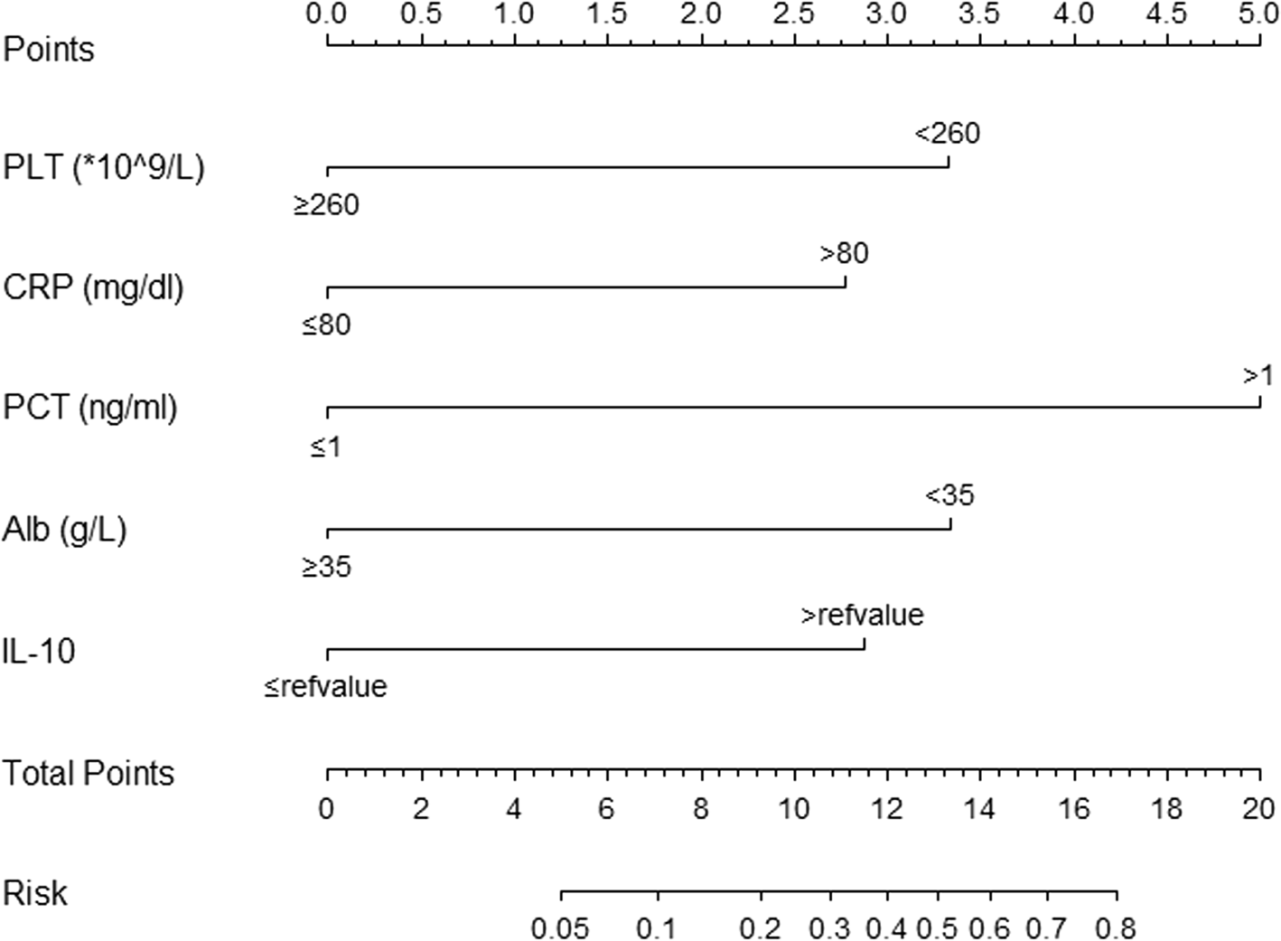
Nomogram for predicting KDSS from KD patients. Abbreviations: KDSS, Kawasaki disease shock syndrome; PLT, platelet; CRP, C-reactive protein; PCT, procalcitonin; Alb, albumin; IL-10, interleukin-10. By summing up scoring points (range: 0–5) from all factors in the nomogram, we can calculate the total points that correspond to the Total Points axis. Subsequently, a vertical line is drawn downwards towards the Risk axis in order to determine the probability of KDSS

For better popularizing in clinical practice, we normalized IL-10 by using the clinical reference values. PLT, CRP, PCT and Alb were transformed into dichotomous variables according to the maximum Youden’s index. The optimal cut-off points are shown in Supplementary Table 2. Incorporating these dichotomous indicators into multivariate logistic regression analysis revealed that IL-10 > reference value, PLT < 260 × 10^9^/L, CRP > 80 mg/ml, PCT > 1 ng/ml, and Alb < 35 g/L were independent risk factors for KDSS [IL-10 > reference value, odds ratio 2.836 (95% CI: 1.346–5.976), *P* = 0.006; PLT < 260 × 10^9^/L, odds ratio 3.343 (95% CI: 1.679–6.658), *P* = 0.001; CRP > 80 mg/ml, odds ratio 2.736 (95% CI: 1.293–5.789), *P* = 0.009; PCT > 1 ng/ml, odds ratio 6.151 (95% CI: 2.818–13.424), *P* < 0.001; Alb < 35 g/L, odds ratio 3.361 (95% CI: 1.581–7.145), *P* = 0.002]. A nomogram visualized the predictive model was constructed (Fig. 1).The score points for PLT < 260 × 109/L, CRP > 80 mg/ml, PCT > 1 ng/ml, and Alb < 35 g/L can be read on the Points per item axis. By summing up scoring points (range: 0–5) from all factors in the nomogram, we can calculate the total points that correspond to the Total Points axis. Subsequently, a vertical line is drawn downwards towards the Risk axis in order to determine the probability of KDSS. For instance, assuming a child with PLT at 310 × 10^9^/L (0 points), CRP at 126 mg/dl (3 points), PCT at 1.2 ng/ml (5 points), Alb 33.9 g/L (3.5 points) and IL-10 > reference value (3 points), the total points were 14.5, the estimated probability amounts to 65%.

### Nomogram performance and validation

**Fig. 2:**
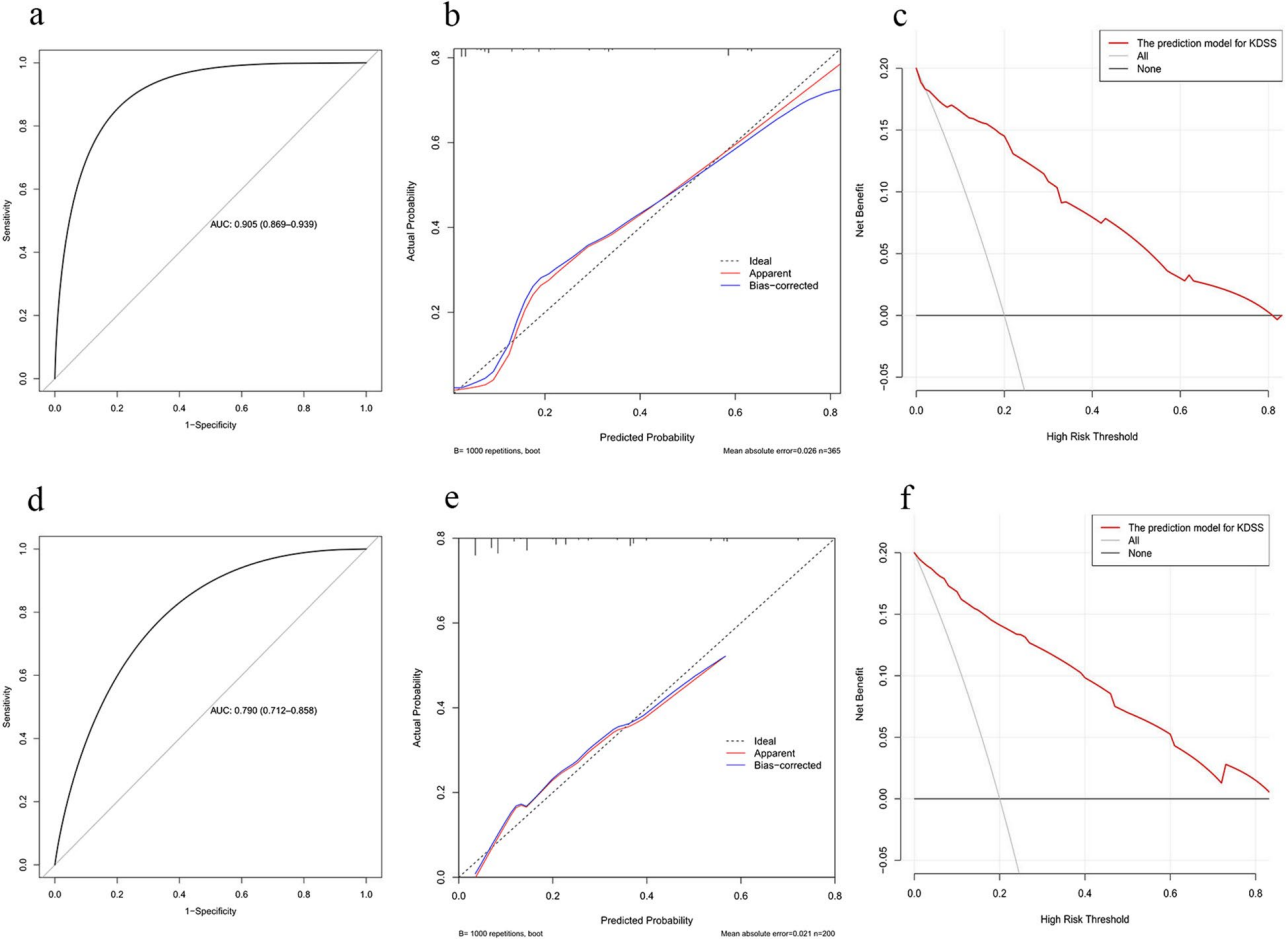
Performance and validation of the nomogram. **a** ROC curve of the nomogram in the development dataset. **b** Calibration curve of the nomogram in the development dataset. **c** DCA curve of the nomogram in the development dataset. **d** ROC curve of the nomogram in the case-independent validation dataset. **e** Calibration curve of the nomogram in the case-independent validation dataset. **f** DCA curve of the nomogram in the case-independent validation dataset

The AUC for the nomogram was 0.91 (95% CI: 0.87–0.94) (Fig. 2a). The specificity and the sensitivity were 80% and 86%, respectively. After 1000 Bootstrap resampling internal validations, the AUC reached as high as 0.89. The calibration curve revealed a good fit during internal validation in patients with a 40%-80% probability of KDSS actually (Fig. 2b).

In the case-independent validation dataset, the model yielded an AUC value of 0.90 (95% CI: 0.71–0.86) with a specificity and sensitivity of 66% and 77%, respectively (Fig. 2d). Figure 2e shows good predictive accuracy of the nomogram in the case-independent validation dataset. The decision curve analyses revealed the predictive model has good application value (Fig. 2c and f).

## Discussion

In this study, we aimed to identify significant risk factors for KDSS and construct a nomogram model to predict KDSS in the early stage of the disease among Chinese children. Our study found that PLT, CRP, PCT, Alb and IL-10 were independently attributable to the onset of KDSS. The nomogram model was established with first-rank discrimination and calibration which would facilitate pediatricians in early diagnosis. To our knowledge, this is the largest study of KDSS by far and the first prediction model for KDSS.

A diagnosis of KDSS was made as if the presence of systolic hypotension or clinical signs of poor peripheral perfusion in KD patients [1]. Children present symptoms including tachycardia, cool and pale extremities, prolonged capillary refill, weak peripheral pulses, mental status change but maintain normal blood pressure in the compensated stage [19]. Since such presentations were also presented in hemodynamically normal KD patients within high temperature, clinicians may miss the diagnosis of KDSS until children go into the decompensated stage with a markable decrease in blood pressure. The predictive model we built can assist clinicians in differentiation between KDSS and hemodynamically normal KD in the early stage, which secondarily influences clinical decision.

Although the etiology of KDSS remains unclear, there has been a widely accepted hypothesis that, in the acute stage of KDSS, severe inflammation induces vascular endothelium dysfunction, which is manifested as increased vascular permeability, and further results in increased protein and water transit to the extra-vascular space [1, 20]. Ma et al. revealed that the patients with KDSS had higher levels of CRP and PCT than the patients with KD [21]. Dominguez et al. found that CRP levels in KDSS patients were on average two-fold higher than those of KD patients [22]. Our study not only found that CRP and PCT were independent risk factors for KDSS, but also found elevated concentrations of IL-10 were independently associated with the risk of KDSS. IL-10, an anti-inflammatory cytokine, has been demonstrated to be involved in autoimmune diseases such as systemic lupus erythematosus and multiple sclerosis [23]. These findings indicated that inflammatory response played an important role in the pathogenesis of KDSS. Current studies have pointed out that genetic susceptibility and different environmental triggers, pathogenic microorganism result in the distinct clinical features of patients with KD [24, 25]. The specific trigger of KDSS needs to be further studied.

Increased PLT is a common feature of KD and contributed to the development of cardiovascular lesions [26]. Thrombocytopenia is relatively uncommon in KD, mostly related to the severe complications of the disease [27]. Our study found that the level of PLT in patients with KDSS was lower than that in the control group, which was the same as reported by most previous studies [28]. Besides, PLT < 260 × 10^9^/L was a significant and independent risk factor of KDSS in this study. Mechanism of thrombocytopenia in KDSS is still unclear, which may be related to abnormally active coagulation mediated platelet consumption [29]. However, due to the lack of data on coagulation indicators before the onset of shock syndrome in this study, the changes in coagulation function need further studies.

In this study, we transformed the value of PLT, CRP, PCT, Alb and IL-10 into dichotomous variables to make the predictive model concision and utility. By using the maximum Youden’s index, we determined the optimal cut-off values for PLT, CRP, PCT and Alb were 260 × 10^9^/L, 80 mg/dl, 1 ng/ml and 35 g/L, respectively. When comparing our results to the previous study, we didn’t apply the ROC curves to find the specific cut-off values of cytokines, but instead used the reference values provided by the respective hospitals to normalize the levels of IL-10. Kaneko. et al. found 363.5 ng/L of IL-6 was accurate for distinguishing between KD [30]. Li et al. demonstrated that 66.7 ng/L of IL-6, 20.85 ng/L of IL-10 can be used to distinguish KDSS from KD [31]. Unlike blood routine examination and biochemical detection, commonly used methods for cytokine detection differed in different institutions and hospitals, including western blot, enzyme linked immunosorbent assay, enzyme linked immunospot assay, chemiluminescent enzyme immunoassay, the human magnetic Luminex assay, reverse transcription-polymerase chain reaction (RT-PCR), the proximity extension assay and so on [32–36]. Besides, there have been multiple manufacturers’ protocols, for instance, R&D Systems, Minneapolis, US; BD Biosciences, California, US; Fuji Rebio Tokyo, Japan. Therefore, the specific cut-off values of cytokines based on the single center were not applicable to other institutions. Using the reference ranges to normalize the cytokines was beneficial to clinical promotion.

This study had several limitations. Firstly, this is a retrospective study conducted in two children’s hospital in Beijing, China, which probably restricted the generalizability of these findings in other regions. Secondly, all patients enrolled in this study was Chinese. The application of this nomogram model in foreign population needs to be verified.

## Conclusion

In conclusion, our research demonstrated that PLT, CRP, PCT, Alb and IL-10 were significantly associated to the risk of KDSS. We constructed a predictive nomogram model with good predictive performances which would facilitate pediatricians in early diagnosis of KDSS and timely intervention in cardiovascular complications.

### Supplementary Information

**Additional file 1: Supplemental Table 1. **Comparison with demographic information and laboratory data between the two groups after propensity score matching in the case-independent validation dataset.

## Data Availability

The datasets generated and/or analyzed during the current study are available from the corresponding author on reasonable request.

## Abbreviations

ANC: Absolute neutrophil count
Alb: Albumin
ALT: Alanine aminotransferase
AST: Aspartate aminotransferase
AUC: Area under the receiver operating characteristic
CRP: C-reactive protein
DCA: Decision curve analysis
ESR: Erythrocyte sedimentation rate
IL-6: Interleukin-6
IL-8: Interleukin-8
IL-10: Interleukin-10
IVIG: Intravenous immunoglobulin
Hgb: Hemoglobin
KD: Kawasaki disease
KDSS: Kawasaki disease shock syndrome
PCT: Procalcitonin
PLT: Platelet
ROC: Receiver operator characteristic curve
RT-PCR: Reverse transcription-polymerase chain reaction
Scr: Serum creatinine level
WBC: White blood cell
