# Supplementary material for: Risk factors and an early predictive model for Kawasaki disease shock syndrome in Chinese children
Source: Ital J Pediatr. 2024 Feb 3;50:22. doi: 10.1186/s13052-024-01597-x (PMC10837898; doi:10.1186/s13052-024-01597-x)
Supplement: Supplementary file 1 — Additional file 1: Supplemental Table 1. Comparison with demographic information and laboratory data between the two groups after propensity score matching in the case-independent validation dataset. [file 13052_2024_1597_MOESM1_ESM.docx]

**Supplemental Table 1. Comparison with demographic information and laboratory data between the two groups after propensity score matching in the case-independent validation dataset**

| Variables | KDSS group (n=40) | KD group (n=160) | P values |
| --- | --- | --- | --- |
| Age, years | 3.58 (2.42, 5.69) | 3.42 (2.5, 5.5) | 0.93 |
| Gender (male) | 26 (65) | 83 (51.88) | 0.19 |
| WBC, ×10^9^/L | 12.88 (9.01, 18.59) | 15.57 (11.5, 18.99) | 0.06 |
| ANC, ×10^9^/L | 11.11 (6.3, 15.41) | 11.04 (7.97, 15.24) | 0.38 |
| PLT, ×10^9^/L | 236.5 (144.75, 333.25) | 290.5 (249.5, 335) | 0.02 |
| Hgb, g/L | 101 (93, 107.25) | 124 (119, 130.25) | <0.001 |
| CRP, mg/L | 102.4 (75.75, 169.25) | 38 (18.98, 69.25) | <0.001 |
| PCT, ng/ml | 1.86 (0.95, 3.60) | 0.53 (0.25, 1.68) | <0.001 |
| ESR, mm/60min | 63.58±31.6 | 66.99±24.54 | 0.46 |
| ALT, U/L | 29.5 (19.5, 54.2) | 23.65 (11.9, 58.5) | 0.12 |
| AST, U/L | 39.65 (26.18, 47.15) | 25.75 (19.35, 35.17) | 0.001 |
| Scr, μmol/L | 27.5 (22.32, 35.92) | 27.95 (23.7, 32.3) | 0.78 |
| Alb, g/L | 28.25 (25.15, 31.18) | 36.3 (33.8, 38.4) | <0.001 |
| IL-6 (>reference value) | 25 (63) | 141 (88) | <0.001 |
| IL-8 (>reference value) | 4 (10) | 152 (95) | <0.001 |
| IL-10 (>reference value) | 5 (13) | 66 (42) | 0.001 |

KDSS, Kawasaki disease shock syndrome; KD, Kawasaki disease; WBC, white blood cell; ANC, absolute neutrophil count; PLT, platelet; Hgb, hemoglobin; CRP, C-reactive protein; PCT, procalcitonin; ESR, erythrocyte sedimentation rate; ALT, alanine aminotransferase; AST, aspartate aminotransferase; Scr, serum creatinine; Alb, albumin; IL-6, interleukin-6; IL-8, interleukin-8; IL-10, interleukin-10. Since the method of detection of cytokines were different in KDSS group and KD group, we used reference ranges to normalize IL-6, IL-8, IL-10. Gender, IL-6, IL-8, IL-10 were expressed as number (percentage). ESR is expressed as mean ± standard deviation, while the other continuous variables are expressed as median with quartile ranges.

**Supplemental Table 2. Refence ranges and cut-off values of selected variables**

| Variables | Reference Range | Optimal cut-off values by Youden's index |  | Selected cut-off value |
| --- | --- | --- | --- | --- |
| PLT, ×10^9^/L | 167-453 | 259.5; 290.5 |  | 260 |
| CRP, mg/dl | <8 | 77; 79; 80 |  | 80 |
| PCT, ng/ml | ≤0.5 | 1.02; 1.04 |  | 1 |
| Alb, g/L | 39-54 | 33.85 |  | 35 |
| IL-10, ng/L | 0-9.1(CIP) |  |  |  |
|  | ≤12.0 (BCH) |  |  |  |

PLT, platelet; CRP, C-reactive protein; PCT, procalcitonin; Alb, albumin. Optimal cut-off values were determined according to the Youden’s index. The reference ranges of IL-10 were 0-9.1ng/L and ≤12.0 ng/L in the Children’s Hospital Capital Institute of Pediatrics, Beijing (CIP) and in the Beijing Children’s Hospital (BCH), respectively.
